# Operative Effect Comparison of Flexible Drill Guiding *vs.* Traditional Drill Guiding Template for Lower Cervical Pedicle Screw Insertion: A Retrospective Analysis

**DOI:** 10.1111/os.13773

**Published:** 2023-06-22

**Authors:** Chao Wu, Jiayan Deng, Haigang Hu, Danwei Shen, Binwei Qin, Xiangyu Wang, Tao Gao, Lian Xu

**Affiliations:** ^1^ Department of Orthopaedics Zigong Fourth People's Hospital Zigong China; ^2^ Institute of Digital Medicine, Zigong Academy of Big Data for Medical Science and Artificial Intelligence Zigong China; ^3^ Department of Orthopaedics Hospital of Southwest Medical University Luzhou China

**Keywords:** 3D Print, Guiding template, Lower cervical, Pedicle screw

## Abstract

**Objective:**

Accurately inserting pedicle screws is the key point of posterior pedicle screw fixation for lower cervical spine (C3–C7) instability. 3D printing technology can improve the accuracy of screw placement. This study compared the safety of 3D‐printed flexible drill guiding template *vs.* traditional rigid drill guiding template for lower cervical pedicle screw insertion.

**Methods:**

This was a retrospective study. A total of 34 patients who underwent lower cervical pedicle screw fixation from March 2018 to May 2021 were enrolled in this study, and they were divided into the flexible drill flexible drill group and the traditional drill group. A total of 18 patients in the flexible drill flexible drill group underwent pedicle screw fixation assisted by 3D printed flexible drill guiding templates for the lower cervix, and 16 patients in the traditional drill group underwent pedicle screw fixation assisted by 3D printed regular drill guiding templates for the lower cervix. The length of the incision and intraoperative blood loss during surgery were recorded and compared for the two groups. The grade, deviation of the screw entry point, deviation of the screw medial angle and screw length were measured and compared after surgery for the two groups by independent‐sample tests.

**Results:**

There was a significant difference in the length of the incision and blood loss between the two groups (*P* < 0.05). There was a significant difference between the two groups for grade (*P* = 0.016). The deviation of the screw entry point was 0.65 ± 0.50 mm in the flexible drill group and 0.78 ± 0.83 mm in the traditional drill group. The deviation of the screw medial angle was 2.14 ± 1.78 in the flexible drill group and 4.23 ± 2.51 in the traditional drill group, with a significant difference between the two groups (*P* < 0.05).

**Conclusion:**

Compared with regular guiding techniques, lower cervical pedicle screw placement assisted by multistep navigation templates and flexible K‐wires results in less trauma and better safety.

## Graphical abstract

(A) The design of multistep expanded flexible drilling templates based on the 3D cervical model, and a virtual screw with a diameter of 3.5 mm was inserted into the pedicle. (B) The K‐wires were inserted assisted by the drill guides based on the 3D printed model. (C) Flexible K‐wires were inserted to determine the pedicle corridor for the pedicle screw in the operation. (D–F) Axial view of C5–C7 after pedicle screw placement, which shows a good position.

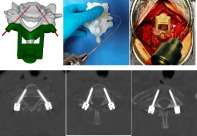

## Introduction

Posterior cervical fixation has become a routine procedure for the treatment of unstable lower cervical spine (C3–C7) due to trauma.^1^, ^2^ Transpedicular screws result in three‐column stability, with biomechanical properties significantly superior to those of lateral masses and laminar screws.^3^, ^4^, ^5^, ^6^ Due to the large morphological difference and complex anatomical structure of each vertebra in the lower cervical spine, the size of the pedicle diameter has individual differences, and it is adjacent to the spinal cord, nerves, vertebral artery and other important structures.^7^, ^8^ Manual manipulation, O‐arm guidance of pedicle screw placement for lower cervical gives an accuracy rate of 55.6% and 70.3%, respectively^9^, ^10^ so accurately inserting the pedicle screw in the lower cervical spine has become an urgent problem to be solved by clinicians.^9^, ^11^

Scholars obtained anatomical parameters of the lower cervical pedicle based on a large number of cadaver anatomical measurements and 3D anatomic analysis, but it is difficult to apply for individuals.^12^, ^13^ Computer navigation can improve the safety and accuracy of screw placement to a certain extent;^14^ however, some shortcomings with these systems persist: drift can greatly affect accuracy, and these errors require modification by adding registration times that could increase the surgeon's radiation exposure and prolong the operation time.^15^ A patient‐specific 3D‐printed drill guiding template, also referred to as the drill guiding template, can assist pedicle screw placement according to individual anatomic differences. In contrast to computer navigation, the drill guiding template has many advantages, such as being independent of vertebral movement, without drifting, providing tactile feedback during drilling, and having no intraoperative fluoroscopy. However, deviation of the screw entry point and screw direction may exist due to the blocking effect of lateral soft tissue for the regular drilling guide template.^15^, ^16^

The pedicle screw of the lower cervical spine differs from that of the upper cervical spine and thoracolumbar spine in that it has a larger medial angle, averaging 43°.^17^ In addition, Mohi Eldin performed anatomic analysis of the lower cervical pedicles of 22 adults and found that the pedicle widths of C3–C7 were 4.3–4.9, 4.6–5, 4.7–5.5, 5.5–6 and 6.0–6.9 mm, respectively.^18^ To improve the safety of pedicle screw placement in the lower cervical spine, researchers have proposed various 3D‐printed navigation templates to assist screw placement, such as combined navigation templates and marked navigation templates.^19^, ^20^, ^21^ Because the drilling is inflexible, a large medial angle and lateral soft tissue tension significantly reduce the accuracy of screw placement assisted by the drill guiding template. The researchers found that the safety rate of lower cervical pedicle screw placement assisted by a 3D‐printed navigation template was 87.5%.^20^ Therefore, the accuracy improvement of lower cervical pedicle screw placement is usually based on more soft tissue dissection and more trauma. In this study, we developed a flexible drill guiding template to assist lower cervical pedicle screw placement, which has not yet been reported. The flexible drill guiding template system is composed of a template with a guiding tube inner diameter of 1.4 mm and a template with a guiding tube inner diameter of 1.7 mm in this research. A flexible 1.2 mm K‐wire was previously used to explore the pedicle screw trajectory along the guide template tube without expanding the incision. After confirming the safety of the entry point, the comparable flexible 1.5 mm K‐wire was replaced to expand the trajectory to form the screw trajectory.

For accurate pedicle screw positioning in the lower cervical spine, researchers have raised different types of 3D‐printed drill guiding templates, and there is still a certain screw placement deviation caused by attaching instability, template sliding, template deformation, low printing accuracy, and so forth.^14^, ^21^ Therefore, the improved screw placement accuracy requires the expense of larger incisions and more dissection of soft tissue.^22^

In this study, multistep expanded guiding templates assisting flexible K‐wire drilling were developed to address these concerns. This multistep expanded guiding templates were composed of a plurality of navigation templates with different inner diameters of guide tubes. This design facilitates the step‐by‐step passage of various K‐wires with different diameters and flexibility. To the best of our knowledge, no study has compared 3D printed flexible drill guiding templates with 3D printed regular drill guiding templates for lower cervical pedicle screw placement. This study aimed to: (i) design multistep expanded flexible drilling templates for lower cervical pedicle screw insertion; and (ii) assess the accuracy and safety of 3D‐printed flexible drill guiding techniques *vs*. traditional rigid drill guiding techniques.

## Materials and Methods

### Patient Population

A total of 34 patients who underwent lower cervical (C3–C7) pedicle screw fixation from March 2018 to May 2021 were enrolled in this study, and all patients signed informed consent for the use of their clinical data. The study was approved by the Ethics committee of Zigong Fourth People's Hospital (2017–04).

Inclusion criteria included lower cervical fracture or instability diagnosed after trauma; lower cervical posterior fixation performed; aged from 20 to 75 years; and followed for more than 1 year. Exclusion criteria included pathological fractures, severe systemic diseases, and severe osteoporosis.

All patients underwent anterior and lateral radiographs and computed tomography angiography (CTA) before surgery. According to the method of drill placement, the patients were divided into the 3D printed flexible drilling template group (Flexible drill group) and the 3D printed regular drilling template group (traditional drill group), and there was no statistical difference in the clinical data between the two groups (Table 1). All the surgeries were performed by the same surgeon. All patients completed outpatient follow‐up 1 month after surgery and telephone follow‐up every 3 months. Each patient completed a total of three follow‐up visits for a total of 13 months.

**TABLE 1:** Patient's demographic data between two groups.

Variables	Flexible drill group (*n* = 18)	Traditional drill group (*n* = 16)	Statistics	*P*
Age (mean ± SD, years)	37.9 ± 9.5	38.2 ± 6.1	1.199	0.231
Gender (*N*)				
Male	12	11	0.017	0.897
Female	6	5		
BMI (kg/cm^2^)	23.5 ± 2.9	22.9 ± 3.1	0.704	0.482
Trauma causes (*N*)				
Motor vehicle accident	9	8	0.018	0.991
High‐energy fall	7	6		
Other injury	2	2		

### Design of Multistep Expanded Flexible Drilling Templates

**Fig. 1:**
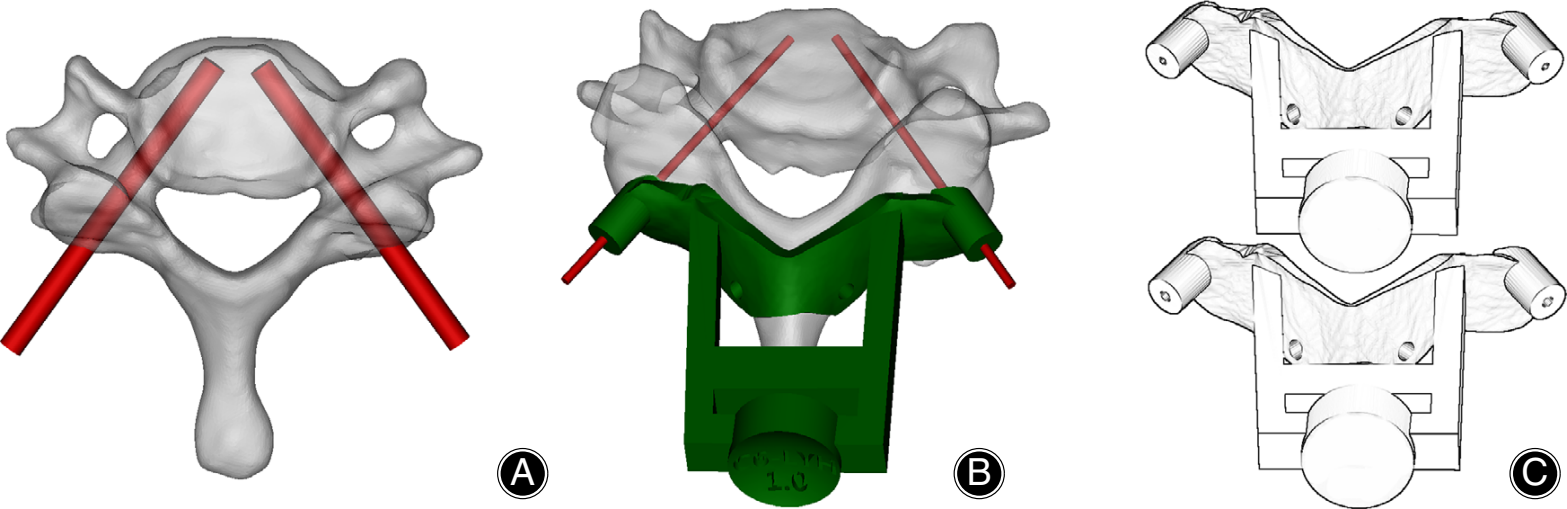
The design of multistep expanded flexible drilling templates based on the 3D cervical model. (A) A virtual screw with a diameter of 3.5 mm was inserted into the pedicle. (B) The drilling templates for pedicle screws were designed. (C) Sketch of drilling templates for 1.2 mm K‐wires and 1.5 mm K‐wires.

The 3D model of the lower cervical spine was reconstructed based on the CT images of bone in Mimics 21.0 (Materialise, Leuven, Belgium), and virtual pedicle screws were placed based on the 3D model (Fig. 1A).The virtual pedicle screw and 3D model of the lower cervical spine were imported into 3‐matics 13.0 (Materialise, Leuven, Belgium). The base of drilling templates was designed to cover the lamina around the screw entry points and the spinous process, and holes with a diameter of 2.7 mm were set on the base for 2.5 mm screws, which can prevent template slippage during operation. A navigation tube with a length of 5 mm was stretched along the direction of the pedicle screw, and inner diameters of 1.3 and 1.6 mm were designed. A handle was designed in an upward direction to allow the surgeon to hold the template accurately. The base, navigation tube with an inner diameter of 1.4 mm and handle were merged to form drill guides for 1.2 mm K‐wire placement. The base, navigation tube with an inner diameter of 1.7 mm and handle were merged to form drill guiding templates for 1.5 mm K‐wire placement (Fig. 1B,C). Guiding templates were imported into the 3D printer (3DS, project 3600; material, photosensitive resin).

### Preoperative Preparation

**Fig. 2:**
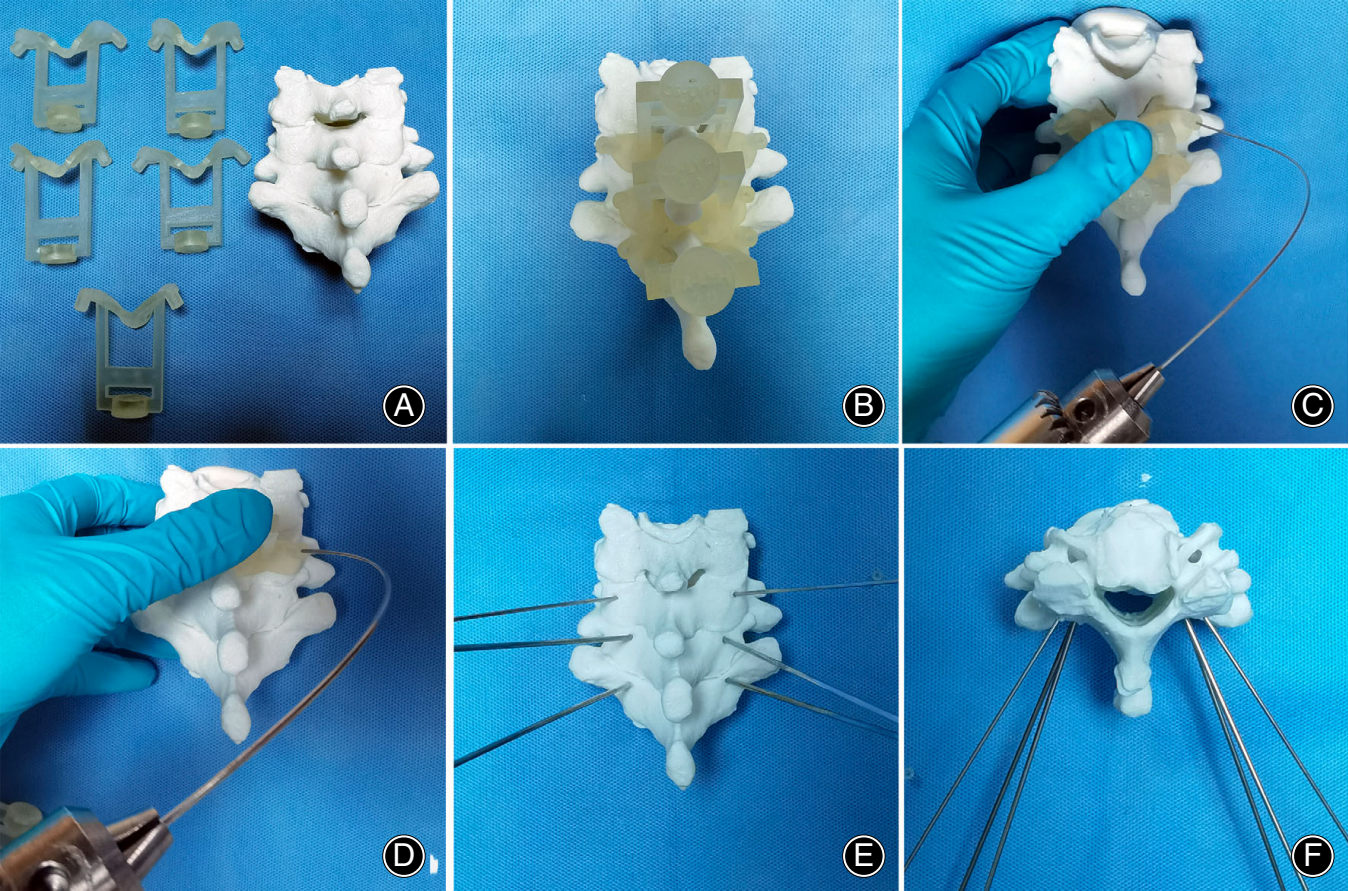
Surgical simulation based on the 3D printed models. (A) The 3D‐printed drilling templates and lower cervical spine. (B) The drilling templates were attached to the model of the cervical spine as designed. (C) The 1.2 mm K‐wires were inserted assisted by the drill guides. (D) The 1.5 mm K‐wires were inserted assisted by the drill guides along the trajectory of 1.2 mm K‐wires. (E, F) Evaluation of the safety and accuracy of the screw trajectory.

The model of the lower cervical spine and guiding templates were made using 3D printing technology (Fig. 2A), and the guiding template was attached to the 3D model (Fig. 2B). In flexible drill group, K‐wires with a diameter of 1.2 mm were first drilled into the pedicle of the corresponding cervical segment under the guidance of a tube with an inner diameter of 1.4 mm, and K‐wires with a diameter of 1.2 mm were easily bent during drilling to reduce the medial angle (Fig. 2C). The position of the entry point and the direction of the K‐wires were observed and confirmed. Since the 1.2 mm K‐wire was previously used as a guide, K‐wires with a diameter of 1.5 mm were easily drilled into the pedicle under the guidance of a tube with an inner diameter of 1.7 mm (Fig. 2D). Finally, the screw was inserted through the K‐wire's trajectory, and the safety of the screw was evaluated (Fig. 2E,F).

### Surgical Technique

Under general anesthesia, the patient was placed in the radiolucent operation table with the prone position, and the head was fixed in a Mayfield head holder.

#### Exposure

**Fig. 3:**
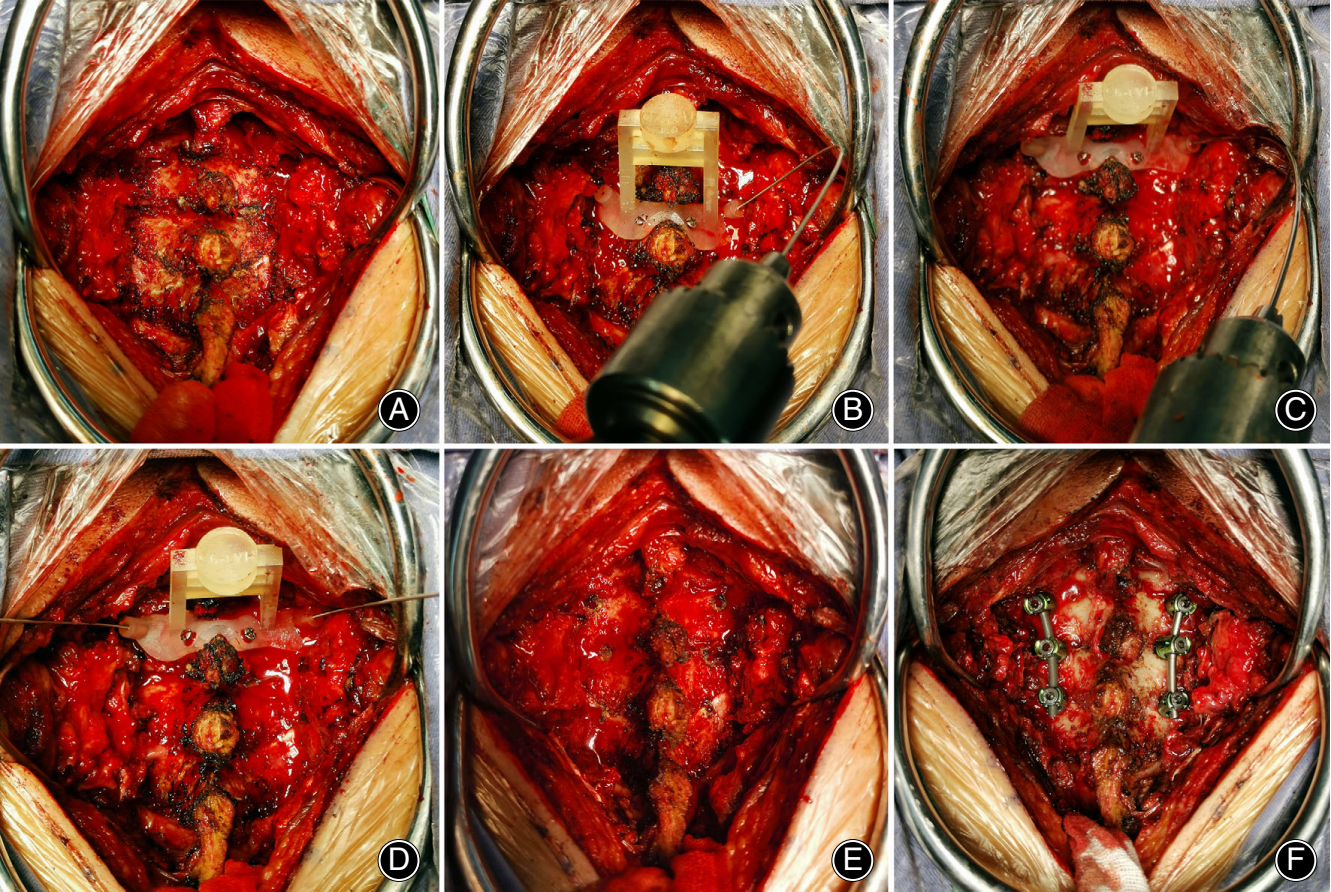
The surgical process of pedicle screw placement assisted by the drilling template. (A) The edge of the lateral mass was exposed sufficiently. (B) The guide templates were completely attached to the lamina and spinous processes and fixed by 2.5 mm screws. Flexible 1.2 mm K‐wires were inserted to determine the pedicle corridor for the pedicle screw. (C) The 1.5 mm K‐wires were inserted to expand the pedicle screw trajectory. (D, E) Confirming the screw trajectory. (F) The 3.5 mm pedicle screws were inserted along the trajectory; the screws were connected and fixed through two connecting rods.

In flexible drill group, the lateral border of the required fixed segment was exposed *via* a posterior median approach (Fig. 3A); the guide base was completely attached to the lamina and spinous processes (Fig. 3B). In traditional drill group, more segments were exposed, and the posterior soft tissue was dissected as laterally as possible to place the 3D printed regular drill guiding template.

#### Preparation of the Screw Path

In flexible drill group, the drill guiding template with an inner diameter of 1.4 mm was first used. When the base of the drill guiding template was completely attached to the bone, fixation was conducted by 2.5 mm screws at the base of the drill guiding template. The easily flexible K‐wires with a diameter of 1.2 mm were used to determine the pedicle corridor (Fig. 3B), and posterior and lateral radiographs of the cervical spine were performed to confirm the location of the screw trajectory. K‐wires and guide were removed, and the corridor was inspected with a probe. After confirming the screw trajectory, the drill guiding template with an inner diameter of 1.7 mm is replaced, and flexible K‐wires with a 1.5 mm diameter are used to expand the 1.2 mm screw trajectory (Fig. 3C,D). For traditional drill group, drilling templates with an inner diameter of 2.2 mm were used to assist the drilling, the diameter of the drill was 2 mm, and it was difficult to bend.^23^

#### Screw Placement and Fixation

The 3.5 mm diameter screws were inserted into the pedicle along the above trajectory, the screws were connected and fixed by two rods (Fig. 3E,F), and the wounds were stratified and sutured.

### Evaluation Criteria

#### Grading (Barrow Neurological Institute, Phoenix, AZ, USA)

All patients underwent CT examination in the lying position 1–3 day after surgery. The examination procedure was performed by the same technician with more than 10 years of experience. Screw positions were observed in axial CT images; Grade A was defined as a screw completely confined within the cortical surfaces, which was regarded as accurate screw placement; Grade B was defined as a transverse foramen breach with a screw obstructing the foramen by 1%–25%, which was regarded as acceptable screw placement; Grades C, D, and E were defined as a transverse foramen breach with a screw obstructing the foramen by 26%–50%, 51%–75% and 76%–100%, respectively; Grade M was defined as a medial breach into the spinal canal, vertebral artery or nerve injury; and Grades C, D, E, and M were regarded as poor screw placement.^24^

#### Neck Disability Index (NDI)

NDI is an important indicator of pain and the ability to of daily life; 0–20 indicates mild dysfunction; 21–40 indicates moderate dysfunction; 41–60 indicates severe dysfunction; 61–80 indicates very severe dysfunction; 81–100 indicates complete dysfunction.

#### Deviation of the Screw Entry Point

The screw entry point deviation between postoperation and preoperation.

#### Deviation of the Screw Medial Angle

The screw medial angle deviation between preoperation and postoperation.

#### Screw Length

The postoperative screw length.

### Statistical Analysis

All statistical analyses were performed in SPSS 19.0 (SPSS Inc.; Chicago, IL, USA). Chi‐square tests were performed for sex, trauma causes, injured segment and screw length between the two groups. Mann‐Whitney‐Wilcoxon tests were performed for grade between the two groups. Independent‐sample *t* tests were performed for age, bosy mass index (BMI), length of the incision, intraoperative blood loss, deviation of the screw entry point, and deviation of the screw medial angle between the two groups. The confidence interval was set at 0.95, and a *P* value less than 0.05 was considered to be statistically significant.

## Results

### Clinical Indications

All the patients underwent surgery successfully, there was no aggravation of nerve injury, and the follow‐up was 12–25 months (average of 17.3 months). No screw loosening or fracture occurred in the two groups. The length of the incision was11.5 ± 2.6 cm in flexible drill group and 17.9 ± 4.6 cm in traditional drill group, with a significant difference between the two groups (*P* < 0.05). The blood loss was 128.9 ± 30.5 mL in flexible drill group and 253 ± 50.7 mL in traditional drill group, with a significant difference between the two groups (*P* < 0.05). The average operation time was 95.7 ± 15.4 min and 132.6 ± 26.5 min for flexible drill group and traditional drill group, respectively, and there is significant difference between two groups (*P* < 0.05); The average hospital staying was 8.6 ± 2.1 and 9.8 ± 3.7 d for flexible drill group and traditional drill group, respectively. There was no significant difference for NDI of 3 months after surgery between two groups (Table 2).

**TABLE 2:** Comparison of perioperative parameters between two groups.

Parameters	Flexible drill group (*n* = 82)	Traditional drill group (*n* = 73)	Statistics	*P*
Screwed cervical				
C3	12	13	0.655	0.957
C4	16	14		
C5	17	12		
C6	19	17		
C7	18	17		
Grade				
A	80	62	−2.417	0.016*
B	2	6		
C	0	5		
D/E/M	0	0		
Deviation of screw entry point (mm)	0.65 ± 0.50	0.78 ± 0.83	−0.713	0.477
Deviation of screw medial Angle (°)	2.14 ± 1.78	4.23 ± 2.51	−5.86	0.000*
Screw length				
Small (10–14 mm)	0	2	3.07	0.216
Medium (16–18 mm)	4	6		
Large (20–28 mm)	78	65		
Length of the incision (cm)	11.5 ± 2.6	17.9 ± 4.6	22.447	0.000*
Intraoperative blood loss (mL)	128.9 ± 30.5	253 ± 50.7	28.067	0.000*
Operation time (min)	95.7 ± 15.4	132.6 ± 26.5	−4.06	0.000*
Hospital staying (d)	8.6 ± 2.1	9.8 ± 3.7	−0.891	0.373
NDI 3 months after surgery	*n* = 18	*n* = 16		
0–20	16	13	0.038	0.846
21–40	2	2		
41–60	0	1		
61–80	0	0		
81–100	0	0		

*Note: p* < 0.05 was considered significant difference.

### Radiological Parameters

A total of 82 screws were inserted in flexible drill group, and 73 screws were inserted in traditional drill group. Twelve screws, 16 screws, 17 screws, 19 screws, and 18 screws were placed in C3, C4, C5, C6, and C7, respectively, for flexible drill group; 13 screws, 14 screws, 12 screws, 17 screws, and 17 screws were placed in C3, C4, C5, C6, and C7, respectively, for traditional drill group.

**Fig. 4:**
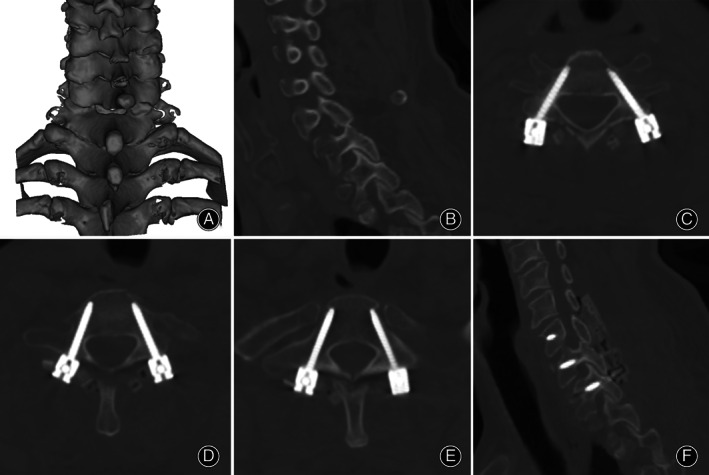
A 45‐year‐old woman was diagnosed with C6 fracture and dislocation with right facet locking. (A) 3D reconstruction of the cervical spine. (B) Sagittal image of the cervical spine shows facet locking. (C–E) Axial view of C5–C7 after pedicle screw placement, which shows a good position. (F) Sagittal image of the cervical spine shows the reduction of facet locking.

There was a significant difference between the two groups for grade (*P* = 0.016, Fig. 4). The deviation of the screw entry point was 0.65 ± 0.50 mm in flexible drill group and 0.78 ± 0.83 mm in traditional drill group. The deviation of the screw medial angle was 2.14 ± 1.78 in flexible drill group and 4.23 ± 2.51 in traditional drill group, with a significant difference between the two groups (*P* < 0.05).

Four screw lengths were 16–18 mm and 78 screw lengths were 20–28 mmin flexible drill group. The two screw lengths were 10–14 mm, 6 screw lengths were 16–18 mm and 65 screw lengths were 20–28 mmin traditional drill group (Table 2).

### Complication

No complications occurred in the flexible drill group; one patient in the traditional drill group developed wound infection and was relieved after dressing change.

## Discussion

In the study, we found that the lower cervical pedicle screw placement assisted by a multistep expansion drill guiding template and flexible K‐wires with less incisions, less blood loss, better safety and more accurate screw placement compared with lower cervical pedicle screw placement assisted by traditional drill guiding. The medial angle of the pedicle screw‐assisted 3D printed drill guiding templates for the lower cervical spine was smaller than the virtual pedicle medial angle.

### Safety and Accuracy of Flexible Drill Guiding Templates

The advantages of this drill guiding template are as follows: first, the incision length and blood loss of flexible drill group were significantly less than those of the traditional drill group. Second, the screw cortical breakthrough rate in the flexible drill group (2.4%, 2/82) was significantly lower than that in the traditional drill group (15.1%, 11/73), which demonstrated the clinical feasibility of lower cervical pedicle screw placement assisted by a flexible drill guiding template system. Third, the length of the majority of the screws in flexible drill group was 20–28 mm (95.1%), which had better mechanical properties than that of traditional drill group (89.0%). Fourth, the medial angle of the pedicle screw‐assisted 3D‐printed drill guiding templates for the lower cervical spine was smaller than the virtual pedicle medial angle. The screw medial angle deviation in the flexible drill group was significantly less than that in the traditional drill group, we think this is a result of soft tissue tension. In the flexible drill group, the flexible drill is used to confirm the safety screw corridor first; then the screw corridor can be expanded step by step under the assistance of guide templates with different tubes; then the spine process can be rotated when screws are inserted to reduce the medial angle of the vertebra, and the screw can be inserted with less soft tissue dissection; fifth, the NDI in the flexible group was significantly lower than that in the traditional drill group, which may be related to the location of the screw and the length of the incision. So, we think the flexible drill guiding template has more advantages in assisting screw placement.

### Surgery Tips

First, minimize the medial angle while ensuring that the screw is located in the safe corridor. Second, the base of the flexible drill guiding templates covers the root of the spinous process, and the screw fixing hole is designed on the base to reduce the risk of base drifting. It is advised to set the guide tube length to 5 mm. Third, the soft tissue of the base‐covered bone surface should be fully stripped out to ensure the stable attachment of the base. Fourth, the depth should be limited when drilling, and the safety of the screw trajectory should be confirmed before replacing the flexible drill guiding templates. Fifth, the spinous process should be slightly rotated to reduce lateral soft tissue tension during screw placement.

### Strengths and Limitation

The strengths of this study include: the length of the wound was reduced and the accuracy was improved by the flexible drill guiding template for lower cervical pedicle screw insertion.

Limitations of this study should be acknowledged. First, patients with lower cervical spine deformities were not included in this study. Second, clinical follow‐up outcomes, including Japanese Orthopaedic Association score and successful fusion, were not investigated in this study. Third, a multicenter study with a large number of patients and long‐term follow‐up is required to validate our findings. Last, different exposure patterns between the two groups brought about other factors that influenced the comparison of clinical outcomes.

### Conclusion

Compared with traditional drill guiding techniques, lower cervical pedicle screw placement assisted by a multistep expansion drill guiding template and flexible K‐wires results in less trauma and better safety.

## Author Contributions

Chao Wu was responsible for the experimental design, paper writing and review. Jiayan Deng for data analysis and paper writing. Danwei Shen and Binwei Qin for data preprocessing. Haigang Hu, Xiangyu Wang, Tao Gao, and Lian Xu for clinical trials.

## Funding Information

This study was supported by Key Project of Zigong Science and Technology (Grant No. 2022ZCYGY04), Sichuan University‐Zigong City Science and Technology Cooperation Project (Grant No. 2021CDZG‐22) and Sichuan Medical Association Research Project (Grant No. 2022SAT04).

## Conflict of Interest Statement

The authors declare that they have no conflicts of interest.

## Ethics Statement

This study was approved by the ethics committee of Zigong Fourth People's Hospital (No. 02, 2013). All patients signed informed consent to participate in the study.

**Consent to Participate:** Informed consent was obtained from all individual participants included in the study.

**Consent for Publication:** The participant has consented to the submission of the case report to the journal. Patients signed informed consent regarding publishing their data and photographs.

## Data Availability

The patients' data and software were authorized to our research.
